# Digitalized “preventive treatment” in chronic disease control and proactive health: a perspective on integrating TCM wisdom with digital intelligence

**DOI:** 10.3389/fdgth.2026.1883420

**Published:** 2026-10-23

**Authors:** Hui Xu, Binyu Luo, Fenfen Hu, Jian Gu, Guilin Shen, Jinchai Xu, Fang Fang, Huiping Qiu

**Affiliations:** 1 Quzhou Hospital of Traditional Chinese Medicine, Quzhou, China; 2 School of Medicine, Quzhou College of Technology, Quzhou, China

**Keywords:** chronic disease prevention and control, digital intelligent health management, non-communicable diseases, preventing a disease before it arises, public health

## Abstract

This Perspective proposes a testable conceptual framework for digitalized preventive treatment that integrates multidimensional data collection, dual-engine analysis, precision health profiling, and personalized intervention. Rather than representing a clinically validated system, the framework organizes existing component-level evidence and defines a pathway for future evaluation of feasibility, effectiveness, safety, equity, implementation, and cost-effectiveness.

## 1 Introduction

Non-communicable diseases (NCDs) have emerged as the primary global public health threat (1, 2). According to the World Health Organization (WHO) report, NCDs account for approximately 71% of all deaths worldwide annually (3). Globally, the extreme disparity in healthcare resource distribution, coupled with the limitations of the traditional “reactive” medical model in addressing the increasingly severe trends of chronic diseases among younger populations and aging societies, has forced a transition from “disease treatment” to “health medicine” (4, 5). This new “prevention-first” paradigm aligns highly with the Traditional Chinese Medicine (TCM) concept of “Preventing a Disease Before It Arises” (Zhi Wei Bing). Its core connotation of “Pre-disease prevention”, “Preventing disease progression”, and “Preventing recurrence” (6, 7) provides theoretical support for longitudinal preventive management across pre-disease and disease-progression-risk stages. However, faced with the dynamic management needs of large-scale high-risk groups, the traditional “Preventing a Disease Before It Arises” model lacks objective quantitative indicators and is limited in service scenarios, making it difficult to achieve continuous and precise risk early warning. This “spatio-temporal limitation” and “data gap” directly constrain the grassroots accessibility and intervention efficacy of TCM preventive services.

In recent years, the rapid development of digital intelligent technology has provided key support for the modern transformation of the concept of “Preventing a Disease Before It Arises” (8). Digital technologies such as the Internet of Things (IoT) and Artificial Intelligence (AI) have enabled new approaches to real-time or repeated monitoring, risk assessment, and digitally supported intervention, although their effectiveness and generalizability vary across applications and settings (9–11). Nevertheless, digital intelligent health management in current practice still exhibits fragmented characteristics of “technology leading, theory lagging,” lacking a deep logical coupling between the TCM holistic view and digital algorithms (12, 13). This Perspective builds on, rather than originates, the broader idea of systems integration between TCM reasoning and biomedical approaches. Its proposed contribution is the digital-health operationalization of these principles into an explicit and testable research architecture that links longitudinal multimodal sensing, biomedical risk assessment, probabilistic TCM constitution–syndrome reasoning, clinical-context fusion, evidence-constrained intervention selection, clinician oversight, and longitudinal feedback. The article does not present a clinically validated digital-health intervention; the feasibility, validity, safety, effectiveness, equity, implementation, and cost-effectiveness of the integrated architecture remain to be prospectively evaluated.

## 2 Definition of concepts

In this paper, high-risk populations primarily refer to two core groups: Sub-healthy populations who possess risks for chronic diseases but have not yet met clinical diagnostic criteria, corresponding to the primary intervention targets of “Pre-disease prevention” within the concept of “Preventing a Disease Before It Arises.” Patients already diagnosed with chronic diseases whose conditions are in the early stages and carry risks of advancement, corresponding to the key management group for “Preventing disease progression.”

Although the broader TCM concept of “Preventing a Disease Before It Arises” also encompasses prevention of recurrence, the operational scope of the present Perspective primarily focuses on individuals at pre-disease risk and patients at risk of disease progression. Recurrence prevention and rehabilitation are acknowledged as part of the broader theoretical continuum but are not presented here as fully operationalized target populations of the proposed architecture.

“Digital intelligent health management” refers to an integrated health service model centered on information technology, covering the entire process of health data collection, analysis, intervention, and evaluation (14). Integration of TCM “Preventing a Disease Before It Arises” and digital technology is conceptualized here as an integrated health-management framework guided by TCM constitution identification and syndrome differentiation thinking, utilizing digital technology to achieve dynamic monitoring of health status, risk early warning, and personalized health cultivation, ultimately forming a model of “TCM wisdom + digital empowerment” (15).

## 3 Theoretical basis

### 3.1 The triple connotations of “preventing a disease before it arises”

The TCM theory of “Preventing a Disease Before It Arises” originates from the core concept in Huangdi Neijing (Yellow Emperor's Inner Canon) that “the sage treats not the already ill but those not yet ill,” encompassing three interrelated and progressively deeper levels. This theory offers explicit guiding value for the precise intervention and interruption of chronic disease progression. Its central emphasis on the principle that “when healthy qi is preserved within, pathogenic factors cannot interfere” highlights the importance of maintaining the balance of yin and yang through holistic body regulation, thereby achieving proactive prevention, control, and risk management of chronic diseases (16).

“Pre-disease prevention” refers to measures targeted at sub-healthy populations who carry risks for chronic diseases but have not yet met clinical diagnostic criteria; it serves as the primary gateway for chronic disease prevention and control. It emphasizes maintaining health balance through diverse means such as mental regulation, rational diet, moderate exercise, and TCM herbal conditioning to fundamentally reduce the risk of disease onset (17).

Within the broader preventive-treatment framework, prevention of disease progression emphasizes early recognition of clinical change, timely management, and syndrome-informed intervention intended to reduce further deterioration and complications (7, 17).

“Preventing recurrence” is directed at populations in the recovery phase of chronic diseases, serving as a vital guarantee for consolidating prevention and control outcomes and avoiding disease recurrence. It focuses on consolidating prevention results through specific constitution conditioning, dietary guidance, and emotional regulation to enhance resistance and avoid recurrence (18).

### 3.2 The logical rationale for digital technology empowering “preventing a disease before it arises”

First, digital technologies may support the operationalization of TCM preventive treatment by facilitating more systematic data collection, longitudinal monitoring, and individualized assessment. The traditional model depends heavily on the personal experience of practitioners and the subjective perception of patients, making it difficult to quantitatively evaluate health status or continuously track intervention effects. Digital technologies can support repeated or continuous collection, storage, and analysis of health-related data, thereby providing additional information for longitudinal preventive management (13, 19). Shang and Zhang (8) discussed the role of digital-intelligent integration in the inheritance and innovative development of TCM. In this context, integrating multimodal physiological data, such as tongue, facial, vocal, and gait features, may support constitution identification and disease-risk prediction and can inform personalized health-management planning. This illustrates a possible route for making elements of pre-disease prevention and prevention of disease progression more quantifiable and traceable, although real-world effectiveness requires further validation.

Second, digital intelligent technology expands the application scenarios of Preventing a Disease Before It Arises and can support health management across settings. Traditional TCM services for Preventing a Disease Before It Arises are mostly limited to medical institutions and constrained by time and space. This makes it difficult to extend these services into daily life, failing to meet the normalized health management needs of high-risk populations. Leveraging the Internet and the Internet of Things (IoT), digital intelligent technology can extend health services to workplaces, homes, and communities. Yu et al. (19) reported an intelligent TCM health management cloud platform using a WeChat mini-program, mobile tablets, and web services, illustrating one practical model for delivering TCM-featured health-management services in daily-life settings.

AI methods have also been used to combine TCM constitution information with biomedical variables for disease-risk prediction, illustrating one possible route for integrating TCM-related features with conventional clinical data (20).

Separately, knowledge-graph and large-language-model approaches have been explored for structuring and retrieving diagnostic reasoning and expert clinical knowledge from TCM medical records (21, 22). These applications support knowledge representation and retrieval, but they do not by themselves establish clinical effectiveness or standardized expert-level decision making.

## 4 Research progress in TCM “preventing a disease before it arises” and digitalized chronic disease control

### 4.1 Risk prediction and early warning

In the identification and early warning of high-risk populations, the deep integration of intelligent TCM technology and the concept of “Preventing a Disease Before It Arises” has demonstrated significant potential. Academician Wang Qi's team established a comprehensive trinity system of “Constitution Identification Theory- Identification Method-Application Mode” by constructing TCM constitution identification theory, establishing new multi-dimensional identification methods, and developing digitalized and intelligent equipment (23). This work reported macro- and micro-level multimodal constitution-identification approaches and linked constitution assessment with disease-risk assessment and health-management functions. These findings illustrate technical feasibility, although external validation, longitudinal stability, and cross-setting generalizability remain insufficiently established. Longitudinal digital phenotyping may support earlier identification and risk stratification, but its incremental clinical value requires prospective evaluation (13).

### 4.2 Early screening and diagnosis

Early screening is critical for implementing “Preventing disease progression.” Professor Shang Hongcai's team utilized “Digital Intelligent TCM Empowering Generalized Inspection” as an entry point, using self-developed equipment for head, face, and whole-body inspection to achieve multi-dimensional, high-precision dynamic capture of human surface signs and biological information (24, 25). This approach was intended to extend the multidimensional capture of macroscopic TCM physical signs beyond conventional episodic assessment. Its incremental diagnostic value, reproducibility, real-world effectiveness, and generalizability require further prospective evaluation. The team reported a prevention-and-control system covering healthy population screening, early warning for target organ damage in hypertension, non-invasive coronary heart disease assessment, and non-perceptual heart failure monitoring. These approaches may provide convenient and non-invasive tools for primary-level screening; their incremental diagnostic value, real-world effectiveness, and generalizability require further prospective evaluation.

### 4.3 Personalized intervention scheme generation

Digital platforms may combine constitution-related information with longitudinal health data to support constitution assessment and prognostic risk estimation. However, prognostic risk alone does not establish whether a particular intervention will benefit an individual. Personalized intervention options should therefore be linked to independently validated intervention-effect evidence, applicable clinical guidelines, and safety constraints; where available, externally validated causal or individualized treatment-effect models may provide additional support for treatment selection. System outputs should remain clinician-reviewable rather than being generated or executed autonomously.

### 4.4 Longitudinal preventive health management practice

Digital technologies have been explored across multiple stages of chronic-disease prevention and management, including risk identification, early intervention, follow-up, and self-management. In the present Perspective, however, the operational focus is on longitudinal management of individuals at pre-disease risk and patients at risk of disease progression. Prevention of recurrence and rehabilitation remain relevant to the broader theoretical connotation of TCM preventive treatment, but they are not treated as fully operationalized target populations within the current architecture.

International digital-health studies have also suggested that multidimensional platforms can improve selected intermediate outcomes such as blood-pressure/glucose control and medication adherence in specific populations (26).

### 4.5 Evidence-based evaluation of intervention effects

Evidence for individual components of digital TCM health management has begun to emerge. Existing studies provide component-level evidence for specific platforms, algorithms, and interventions; however, the effectiveness of the integrated architecture proposed in this Perspective has not yet been established. Qin et al. (27) conducted a clinical controlled study on the intervention effects of TCM artificial intelligence technology for populations at high risk for and patients with type 2 diabetes mellitus (T2DM). Follow-up results after 12 months of intervention showed that blood pressure, blood glucose, and blood lipid levels in both groups decreased significantly compared to pre-intervention, with the observation group showing significantly greater reductions across all indicators. Furthermore, diabetes awareness and self-management efficacy in both groups were markedly improved, with the observation group scoring significantly higher, providing preliminary evidence-based support for the effectiveness of the TCM-AI health management model in chronic disease control. Existing systematic reviews of digital health interventions for hypertension have primarily evaluated outcomes such as blood-pressure control, lifestyle behaviours, and medication adherence rather than long-term hard clinical endpoints (28). More broadly, the studies discussed in this Perspective provide limited evidence on major cardiovascular events, disability, or long-term mortality, underscoring the need for longer-term prospective evaluation. Future studies should evaluate feasibility (recruitment, adherence, and data completeness), algorithmic performance (discrimination, calibration, uncertainty, and external validation), clinical and patient-reported outcomes, implementation outcomes, safety, equity, and health-economic outcomes through multicenter prospective designs.

### 4.6 Systematic innovation of integration mechanisms

The deep integration of TCM “Preventing a Disease Before It Arises” and digital intelligent technology is reflected in mechanistic innovations across service processes, decision-making logic, and governance systems. Digital technologies may support a shift from intermittent assessment toward more continuous and proactive management. Through the synergy of 5G, wearable devices, and AI, a closed-loop system of “data collection—risk assessment—intervention implementation—effect tracking” can support more continuous health management across settings (29). The coordination of TCM reasoning and AI algorithms may support a human–machine collaborative decision-making model. Using classical theories as a framework, the “holistic view” and “syndrome differentiation and treatment” could be represented within computational correlation networks. Deep learning applied to multimodal data may support a proposed workflow linking theoretical representation, multimodal perception, explainable decision support, and clinician-supervised implementation (30). In local practice, a TCM chronic-disease management model has been implemented through online-offline interaction and a care-linkage network, illustrating one approach to coordinated, continuous care (31).

## 5 Theoretical construction of a testable conceptual architecture for digitalized preventive treatment

In recent years, although digital intelligent health management has achieved certain results in chronic disease prevention and control, current practices often focus on single technical advances. However, existing approaches often remain fragmented and do not consistently operationalize TCM holistic reasoning and digital algorithms within a unified, prospectively testable digital-health architecture. Based on the preceding evidence and theoretical rationale, we propose a testable conceptual architecture for digitalized preventive treatment (Figure 1). The purpose of this architecture is not to claim systems integration itself as novel, but to make the proposed integration operationally explicit and empirically testable by specifying the data flow, parallel inference pathways, clinical-context fusion, intervention-evidence constraints, human oversight, and feedback mechanisms. The framework is therefore presented as a research model rather than as an established or clinically validated system.

**Figure 1:**
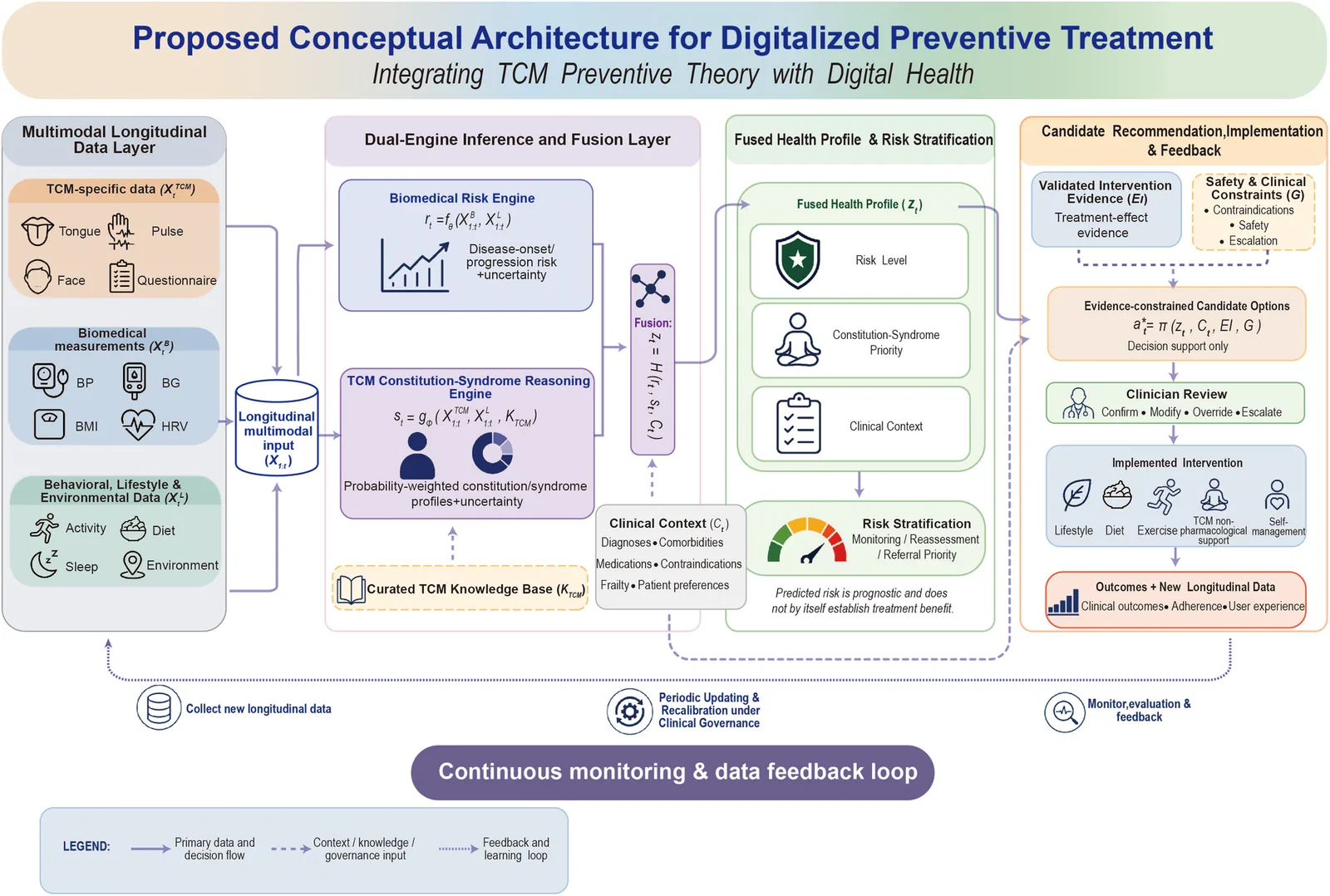
Proposed conceptual architecture for digitalized preventive treatment. Multimodal longitudinal data comprise TCM-specific data ($X_{t}^{\mathrm{TCM}}$), biomedical measurements ($X_{t}^{B}$), and behavioral, lifestyle, and environmental data ($X_{t}^{L})$. Two parallel inference pathways generate biomedical disease-onset/progression risk with uncertainty, $r_{t}=f_{\theta }{(X}_{1:t}^{B}{,X}_{1:t}^{L})$, and probability-weighted TCM constitution–syndrome profiles with uncertainty, $s_{t}=g_{\phi }{(X}_{1:t}^{\mathrm{TCM}}{,X}_{1:t}^{L},K_{\mathrm{TCM}})$, informed by a curated TCM knowledge base ($K_{\mathrm{TCM}}$). Their outputs are integrated with the current clinical context ($C_{t}$) to form a fused health representation, $z_{t}=H(r_{t},s_{t},C_{t}$), which supports risk stratification and management prioritization. The biomedical output *r_t_* is a prognostic estimate used for risk stratification and for prioritizing monitoring, reassessment, or referral; it is not interpreted as evidence that a specific intervention will benefit the individual. Candidate intervention options are considered only after incorporating an independent intervention-evidence layer, *E_I_*, which may include validated guideline-supported treatment effects and, where available, externally validated causal or individualized treatment-effect models. A candidate option can therefore be represented as a_t_* *=* *π(z_t_, C_t_, E_I_, G)*, where *G* denotes safety, contraindication, and escalation constraints. All clinically consequential options require clinician review before implementation. Clinical outcomes and newly collected longitudinal data subsequently feed back into the system for monitoring, updating, and recalibration under clinical governance. Solid arrows indicate primary data and decision flow, dashed arrows indicate contextual, knowledge, or governance inputs, and dotted arrows indicate feedback and learning loops. The figure represents a testable conceptual architecture rather than a clinically validated system.

### 5.1 From “point-in-time assessment” to “longitudinal digital phenotyping”: supporting longitudinal monitoring of the “pre-disease” state

Traditional TCM “Preventing a Disease Before It Arises” relies on the four examinations conducted by practitioners at specific points in time, obtaining instantaneous syndrome information that makes it difficult to capture the dynamic evolution of disease occurrence and development. Digital phenotyping technology, through wearables, smartphone sensors, and multi-modal data fusion, achieves continuous and passive data collection across individual behavioral, physiological, psychological, and environmental dimensions. Research (32) has illustrated how deep phenotyping that combines continuous glucose monitoring, sleep tracking, and multi-omics data can characterize changes along the health-disease continuum. In the present framework, digital phenotyping may extend observation beyond episodic clinical encounters and support earlier identification and risk stratification; its incremental predictive and clinical value remains subject to prospective validation.

### 5.2 From “population management” to “precision constitution-based health cultivation”: supporting individualized intervention

The TCM concept of “treatment based on the individual” emphasizes the core role of constitution differences in health management. Academician Wang Qi's TCM constitution theory provides the theoretical framework for individualized intervention. However, in the traditional model, “constitution identification” is highly dependent on the practitioner's experience, making “health cultivation” difficult to scale. The introduction of machine learning technology is changing this landscape. The TCM-DS large language model developed by Li et al. (33) automatically generates personalized medicinal and edible homology formulas based on symptom and constitution data and reported a recommendation precision of 0.9924 for the specific recommendation task and dataset evaluated in that study. This result provides evidence of task-level technical performance, whereas its clinical effectiveness and generalizability remain to be established. Its supporting platform enables automated constitution analysis and personalized scheme recommendation, illustrating the potential shift of “constitution-based health cultivation” from an experience-based “one-size-fits-all” model to a data-driven model. By utilizing algorithms for refined clustering of different constitution groups, targeted prevention advice can be provided for high-risk individuals, but prospective external validation is required before application to high-risk populations.

### 5.3 From “experience-driven practice” to a “dual-engine fusion” architecture: supporting the inheritance of expert knowledge

The diagnostic and treatment experience accumulated over thousands of years in TCM, especially the wisdom of renowned experts in syndrome differentiation and treatment, is a valuable asset for chronic disease control. However, this knowledge has long existed in the form of implicit experience, making it difficult to replicate and promote. The fusion of knowledge graphs and large language models (LLMs) provides a technical path to solve this problem. Duan et al. (21) developed a TCM case-based question-answering system using a knowledge graph and LLM-assisted extraction methods, and reported favorable fidelity and relevance in the study-specific evaluation. These results demonstrate task-level technical performance rather than externally validated clinical effectiveness. The TCM-KLLaMA study (22) reported lower hallucination-related errors and better performance than the evaluated baseline models on formula-generation tasks after incorporating knowledge-graph and knowledge-injection mechanisms. Whether these improvements generalize to clinical decision support or patient outcomes remains uncertain. These studies illustrate how expert knowledge can be structured and retrieved to support the TCM reasoning component of a digital health architecture. In the framework proposed here, the dual-engine architecture refers specifically to the parallel biomedical risk-assessment and TCM constitution–syndrome reasoning pathways described below, rather than to an autonomous diagnostic or prescribing system. The three conceptual transitions developed across Sections 5.1–5.3 are summarized in Figure 2.

**Figure 2:**
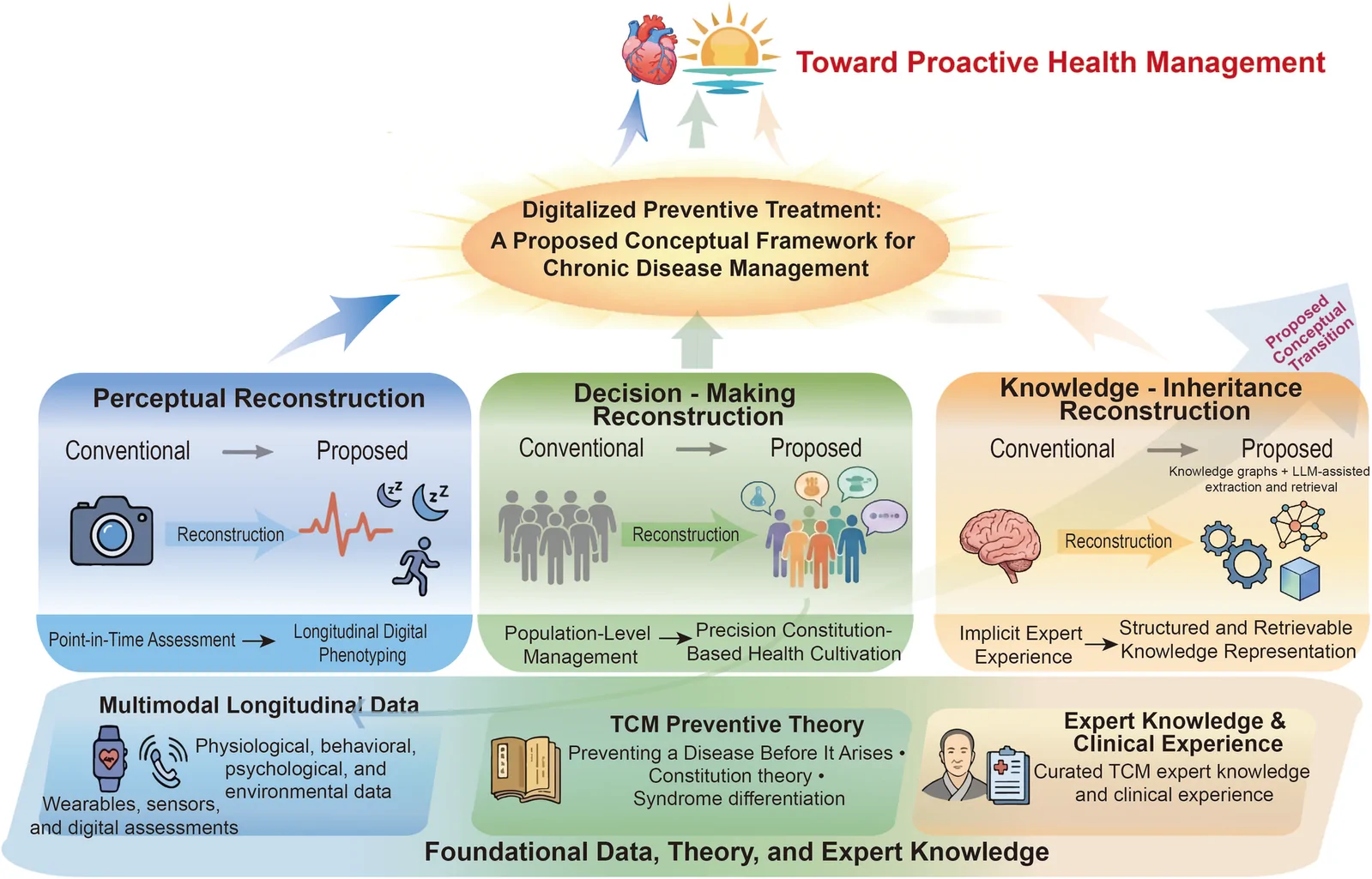
Proposed conceptual transitions underpinning digitalized preventive treatment. The figure summarizes three interrelated transitions from conventional approaches to the proposed digitalized framework: (1) from point-in-time assessment to longitudinal digital phenotyping in the perceptual dimension; (2) from population-level management to precision constitution-based health cultivation in the decision-making dimension; and (3) from implicit expert experience to structured and retrievable knowledge representation in the knowledge-inheritance dimension. These transitions are grounded in multimodal longitudinal data, Traditional Chinese Medicine (TCM) preventive theory, and curated expert knowledge and clinical experience. Knowledge graphs and large language model (LLM)-assisted extraction and retrieval are proposed as tools for structuring and accessing expert knowledge. Together, these elements support a conceptual framework for chronic disease management oriented toward proactive health management. The figure is conceptual and does not imply that the proposed transitions or their clinical benefits have been empirically validated.

### 5.4 Operationalization of the dual-engine fusion architecture

To make the proposed architecture operationally explicit, we define two parallel inference pathways rather than treating the “dual-engine” concept as an opaque computational module. At each time point *t*, multimodal observations are represented as $X_{t}={[X}_{t}^{B}{,X}_{t}^{\mathrm{TCM}}{,X}_{t}^{L}]$, where $X_{t}^{B}$ denotes biomedical measurements, $X_{t}^{\mathrm{TCM}}$ denotes TCM-specific perceptual and symptom data, and $X_{t}^{L}$ denotes behavioral, lifestyle, and environmental information. Their longitudinal histories up to time *t* are denoted by $X_{1:t}^{\;B},\;X_{1:t}^{\mathrm{TCM}}$, and $X_{1:t}^{\;L}$, respectively.

The biomedical risk engine would generate $r_{t}=f_{\theta }{(X}_{1:t}^{B}{,X}_{1:t}^{L})$ representing estimated disease-onset or progression risk and associated uncertainty. In parallel, the TCM reasoning engine would generate $s_{t}=g_{\phi }{(X}_{1:t}^{\mathrm{TCM}}{,X}_{1:t}^{L},K_{\mathrm{TCM}})$, representing probability-weighted constitution and syndrome profiles with associated uncertainty, informed by a curated TCM knowledge base $K_{\mathrm{TCM}}$.

The fused representation *z_t_* is used primarily to characterize current health state, prognostic risk, and management priority. Importantly, the prognostic estimate *r_t_* answers the question of who is at higher risk; it does not establish who will benefit from a particular intervention. Treatment selection therefore requires a separate intervention-evidence input, *E_I_*, representing validated intervention effects from applicable clinical guidelines and empirical studies and, where available, externally validated causal or individualized treatment-effect models. A candidate intervention option can be expressed as a_t_* *=* *π(z_t_, C_t_, E_I_, G)*, where *G* represents safety rules, contraindication screening, uncertainty thresholds, and predefined escalation criteria. In the absence of sufficiently validated intervention-effect evidence, an elevated predicted risk may justify intensified monitoring, reassessment, shared decision-making, or referral, but should not by itself generate a specific treatment recommendation. Clinically consequential options would require clinician review, with explicit mechanisms for confirmation, modification, override, or escalation.

Subsequent outcomes and newly collected longitudinal data would feed back into the system for periodic model updating and recalibration under clinical governance. Importantly, these equations are intended only as a formal representation of the proposed information flow rather than as fitted, trained, or clinically validated models or decision policies. The functional forms and parameters represented by $f_{\theta }$ and $g_{\phi }$, together with the fusion function *H* and recommendation policy π, remain to be specified and, where data-driven, trained. The resulting architecture would require external validation and prospective evaluation before clinical implementation.

### 5.5 Case vignette: digitalized management of “preventing a disease before it arises” for prediabetes

To illustrate the synergistic operation of the triple theoretical reconstructions, this section presents a simulated application scenario for a prediabetic individual based on clinical guideline logic and literature evidence (Box 1).

Box 1 Simulated vignette of the dual-engine workflow.

**Table:** 

Step	Illustrative content and governance
Simulated profile	A hypothetical 45-year-old man with BMI 27.5 kg/m2, fasting blood glucose 6.3 mmol/L, HbA1c 6.1%, a postprandial CGM peak of 9.8 mmol/L, and sedentary time exceeding 10 h/day. These values are illustrative inputs, not observed study data.
Biomedical engine	Longitudinal glucose, anthropometric, activity, sleep, and other available clinical data are used to estimate prediabetes progression risk and uncertainty. Any output requires calibration and external validation.
TCM reasoning engine	Tongue, pulse, facial, symptom, and questionnaire data are combined with a curated TCM knowledge base to generate a probabilistic constitution-syndrome profile with confidence information, rather than a deterministic label.
Fusion and context	The two outputs are reconciled with medical history, current medication, contraindications, patient preferences, and applicable clinical guidance.
Candidate plan	The system may present clinician-reviewable options for diet, physical activity, sleep, self-monitoring, and, where supported by applicable evidence and clinical guidance, appropriate TCM non-pharmacological support. These options are not inferred from predicted progression risk alone. The prognostic output is used to inform monitoring and management priority, whereas intervention selection must be supported by intervention-effect evidence, safety constraints, clinical context, and clinician judgment. The system does not diagnose, prescribe, or execute recommendations autonomously.
Human review and safety	A clinician verifies risk level, checks contraindications, reviews uncertainty flags, explains options, and escalates abnormal glucose, blood pressure, or symptoms according to predefined rules.
Feedback and evaluation	At follow-up, the system records adherence, missingness, adverse events, patient-reported outcomes, glucose and weight trends, clinician overrides, and implementation burden. These are outcomes to be monitored, not claimed treatment effects.

Illustrative only; not an actual patient report, treatment-effect claim, or validation study.

## 6 Main challenges

### 6.1 Data silos and security risks

In the process of practical implementation, data silos remain a significant bottleneck because data collected from medical information systems, wearable devices, and personal health records belong to different management entities and systems. This fragmentation restricts the large-scale utilization of health data and the realization of its integrated value (34). Simultaneously, privacy and security issues regarding health data persist. TCM health data contains not only basic biomedical information but also personal characteristic information such as tongue signs, pulse signs, and constitution types. Any data breach could lead to privacy concerns or even trigger ethical controversies (35). Therefore, a key prerequisite for deepening the integration is achieving a precise balance between promoting data sharing and strengthening privacy protection. This requires governance and technical mechanisms that minimize unnecessary transfer or exposure of identifiable health data (36). Privacy-preserving approaches such as federated learning may support distributed analysis in selected settings, but they do not replace requirements for consent, access control, security testing, auditability, and institutional accountability (35, 36).

Operational governance should include data minimization and purpose limitation; transparent, tiered consent and withdrawal mechanisms where applicable; role-based access control; encryption in transit and at rest; audit trails and data provenance; defined retention and deletion schedules; and explicit allocation of responsibilities across institutions. Where appropriate, federated learning or other privacy-preserving computation may reduce unnecessary transfer of identifiable data, although such techniques do not eliminate the need for governance, security testing, and accountability.

### 6.2 Clinical applicability, generalizability, and algorithmic bias

Currently, existing TCM intelligent intervention and evaluation models face limitations in terms of applicability and practicality. First, the training data for these models mostly originates from a single medical institution or specific populations, resulting in a narrow sample coverage and weak generalization ability (37). Second, the model designs are not closely integrated with actual clinical workflows. There is an overemphasis on enhancing technical performance while neglecting the usage habits of clinical medical staff, which increases their operational burden and reduces application adherence (38). Consequently, enhancing the clinical applicability of intelligent models and increasing the level of acceptance among medical staff are crucial for the practical implementation and effective functioning of digital intelligent health management.

Bias may arise from single-center sampling, regional differences, age or sex imbalance, socioeconomic status, device variability, patterns of digital engagement, and variability among TCM labelers. Evaluation should therefore report subgroup sensitivity, specificity, and calibration; perform external validation across institutions and devices; document inter-rater uncertainty for TCM labels; and include post-deployment drift monitoring and periodic fairness audits. A high aggregate accuracy should not substitute for subgroup performance and calibration assessment.

### 6.3 Digital literacy and acceptance of primary healthcare personnel

The insufficient digital literacy and low acceptance of digital intelligent health management among primary healthcare practitioners constitute another bottleneck restricting its popularization at the grassroots level. First, primary medical staff have limited understanding and mastery of new technologies such as Artificial Intelligence and digital intelligent monitoring, making it difficult to fully utilize intelligent auxiliary tools in areas like constitution identification, risk early warning, and intervention guidance. Second, some primary medical staff remain skeptical, fearing that intelligent technology might replace or weaken their professional judgment—a state of “technology anxiety” that leads to a lack of initiative in adopting new technologies (39). Third, elderly patients, who are the primary group for chronic disease management, generally face a “digital divide.” They encounter obstacles in operating intelligent monitoring equipment and online health management platforms, making it challenging for them to cooperate with digitalized health management processes (40).

Mitigation should include accessible interfaces, large-font and voice interaction, assisted-use pathways, offline or non-smartphone alternatives, and support from community health workers or caregivers. Lack of digital access or literacy should not become a new eligibility barrier; equivalent non-digital routes to assessment and follow-up should remain available.

### 6.4 Lag in standardization of TCM perceptual data

With the wide application of digitalized TCM equipment, the standardization of perceptual data has become increasingly prominent, serving as a key bottleneck restricting the promotion of fusion models. First, TCM diagnostic information has long relied on the subjective experience of practitioners, consisting primarily of qualitative descriptions. The lack of unified terminology—where multiple expressions often exist for the same symptom—makes it difficult to meet the requirements of Artificial Intelligence for high-quality annotated data (41). Second, tongue and pulse image acquisition devices developed by different manufacturers lack unified specifications for technical indicators such as light source environments, sensor parameters, and image resolutions (42). Research indicates that inconsistent lighting conditions and color card standards lead to significant differences in image color reproduction, directly affecting the accuracy of subsequent analysis and data mutual recognition (43). The World Federation of Chinese Medicine Societies has initiated an international standard for TCM tongue-image data exchange, underscoring the need for interoperable acquisition and transmission standards (44).

### 6.5 Clinical safety and human oversight

The architecture requires predefined escalation and referral rules for abnormal blood pressure, glucose, new symptoms, or other safety signals; contraindication screening; transparent communication of uncertainty; and documented clinician review and override. LLM-supported functions should use curated knowledge sources, retrieval grounding, source provenance, output logging, and audit procedures. The Virtual General Practitioner metaphor does not authorize autonomous diagnosis or prescribing, and professional accountability must remain explicit.

### 6.6 Health-economic uncertainty and cost-effectiveness

No evidence currently establishes the cost-effectiveness of the complete architecture. Future evaluations should capture device, software, platform, training, maintenance, clinician time, patient time, and healthcare-utilization costs alongside outcomes. Cost-effectiveness analysis, cost-utility analysis, and budget-impact analysis should be prespecified where appropriate, with reporting of incremental cost-effectiveness ratios and cost per quality-adjusted life-year when the design and jurisdiction support such estimates.

## 7 Future prospects

### 7.1 Regional data platforms and privacy computing technology

To address the prominent issue of data silos, future efforts should focus on constructing secure, efficient, and interoperable regional TCM health data-sharing platforms to break down data barriers and release the value of data fusion. Regarding the enhancement of data privacy protection, cutting-edge technologies such as privacy computing should be actively introduced to build a secure sharing mode characterized by “data stays while models move, and data is available but not visible,” thereby ensuring personal privacy and security. Simultaneously, a transparent and controllable data authorization mechanism and security supervision system should be established.

By clarifying the scope, permissions, and processes of data use, users can clearly understand how their health data is utilized and retain the right to be informed and control its use, thus building a robust “firewall” for data security.

### 7.2 Lightweight intelligent equipment and primary-level adaptation

In response to the insufficient clinical applicability of intelligent models and the difficulties of promotion at the grassroots level, future development should focus on lightweight intelligent equipment and supporting services that match the capacity of primary healthcare and the operational skills of primary medical staff. This will facilitate the downward extension of technology to the grassroots level. Meanwhile, targeted training materials, operation manuals, and technical support systems should be developed to establish an integrated “equipment + training + after-sales support” solution, comprehensively enhancing the equipment utilization capabilities of both primary medical staff and users, and ensuring that technology takes root at the grassroots level.

### 7.3 Standard systems and data mutual recognition

To address the lag in the standardization of TCM perceptual data, future work should strive to construct a technical standard system covering the entire chain of “collection–transmission–storage–analysis.” At the same time, the formulation of unified technical specifications for four examinations data acquisition equipment must be accelerated. For key diagnostic indicators such as tongue signs, pulse signs, and facial complexion, minimum standards for core indicators—including light source environments, sensor parameters, and image resolutions—should be clearly defined.

Establishing equipment admission and calibration mechanisms may improve the comparability and mutual recognizability of data collected by devices from different manufacturers. A systematic TCM perceptual-data standards system may improve interoperability, comparability, and cross-platform data use if accompanied by validated standards and governance arrangements (44).

### 7.4 Staged validation and implementation pathway

Validation should proceed in stages. Stage 1 should establish data quality, measurement agreement, discrimination, calibration, uncertainty estimation, and external validity across sites, populations, and devices. Stage 2 should use feasibility and implementation pilots to assess recruitment, retention, adherence, usability, data completeness, workflow burden, clinician overrides, and adverse events. Stage 3 should evaluate clinical effectiveness and implementation through multicenter pragmatic or cluster-randomized studies with adequate follow-up for patient-reported, clinical, safety, equity, and economic outcomes. Progression between stages should depend on prespecified performance and safety criteria.

## 8 Discussion

Integrating the TCM principle of Preventing a Disease Before It Arises with digital health is relevant to the broader aim of reducing premature mortality from non-communicable diseases (45). The present work frames this integration as a testable research architecture rather than an established paradigm shift. Its value depends on whether future studies demonstrate acceptable feasibility, algorithmic validity, safety, clinical effectiveness, equity, implementation, and cost-effectiveness.

Contextual mapping against related conceptual frameworks and implemented digital interventions helps clarify the scope of the proposal. These comparators differ substantially in conceptual level, clinical purpose, implementation status, and evidence maturity; they are therefore presented to locate the current architecture within the existing landscape rather than as equivalent forms of evidence or as direct head-to-head comparators. Smart TCM describes a broad technology ecosystem spanning prevention, screening, diagnosis, treatment, and management in primary care, while acknowledging constraints related to data, applicability, and digital literacy (46). AI-HEALS is described in a published protocol as a WeChat-based health-education and self-management platform for type 2 diabetes, incorporating knowledge-base question answering, health-data recording, reminders, personalized messaging, and planned economic evaluation; it does not include a reported TCM constitution component (47). The SMART Diabetes program provides cluster-randomized evidence for a multifaceted digital intervention in primary care (9), but it does not test TCM constitution-syndrome reasoning. These examples differ in purpose, clinical scope, and evidence maturity and should not be treated as interchangeable.

Two additional conceptual antecedents help define the boundary of the present proposal. Wei et al. proposed a complex-systems evidence framework for Chinese medicine that organizes multi-source evidence into a four-phase loop of production, differentiation, application, and validation, with emphasis on individualized decision-making and real-world feedback (48). Gou et al. described the state-target strategy as a methodological bridge between TCM state-based reasoning and target-oriented biomedical treatment (49). These approaches demonstrate that systems integration, individualized reasoning, and the conceptual bridging of TCM and biomedical medicine are not novel in themselves. The present Perspective is more narrowly distinguished by its digital-health operationalization of related principles into a specified longitudinal data flow, parallel biomedical and TCM inference pathways, explicit separation of prognostic risk from intervention-effect evidence, clinician-governed decision making, and feedback/recalibration mechanisms.

The incremental contribution of this Perspective lies neither in proposing the first TCM-AI system nor in claiming that systems integration itself is novel. Related conceptual frameworks have already emphasized systems thinking, individualized decision-making, integration of TCM and biomedical reasoning, and iterative feedback. The narrower contribution proposed here is to operationalize these principles within an explicit digital-health architecture that specifies longitudinal multimodal sensing, parallel biomedical and TCM inference pathways, clinical-context fusion, evidence-constrained intervention selection, clinician oversight, and longitudinal feedback and recalibration. Table 1 summarizes these distinctions while making clear that the integrated architecture remains unvalidated.

**Table 1:** Contextual comparison of conceptual antecedents, implemented digital interventions, and the proposed digital-health architecture.

Framework	Framework/intervention type	Primary purpose	TCM component	Digital/AI component	Evidence status	Key comparison point
Smart TCM (46)	Broad conceptual/technology ecosystem	Whole-chain primary-care support for major chronic diseases	Broad TCM prevention, screening, diagnosis, treatment, and management	Portable devices plus generative and predictive AI	Narrative review and emerging component-level evidence	Broad TCM–AI ecosystem; does not validate the proposed architecture.
Complex-systems evidence framework (Wei et al., 2026) (48)	Conceptual/theoretical framework	Organize multi-source TCM evidence through a four-phase loop of production, differentiation, application, and validation	Holistic and pattern-based diagnosis and treatment; integration along the disease-pattern-formula evidence axis	Digital/AI components are enabling methods rather than a specific digital-health intervention	Conceptual framework/editorial; not a validated digital intervention	Conceptual precedent for evidence integration and feedback; no specified longitudinal dual-engine digital architecture.
State-target strategy (Gou et al., 2021) (49)	Conceptual/theoretical therapeutic strategy	Bridge TCM state-based reasoning with target-oriented biomedical treatment	Integrates state identification with target selection to connect TCM reasoning and biomedical treatment targets	No inherent digital/AI implementation component	Conceptual methodological article; not an implemented digital intervention	Bridges TCM reasoning and biomedical targets without specifying a longitudinal digital-health architecture.
Original AI-HEALS (47, 50)	Implemented disease-specific digital intervention/study	Health education and self-management for type 2 diabetes	No TCM-specific constitution component reported in the original AI-HEALS design	WeChat-based knowledge-base Q&A, health-data recording, reminders, and personalized messages	Cluster-randomized mixed-methods protocol (47); qualitative user-experience evidence (50)	Digital diabetes self-management without explicit biomedical-risk and TCM-reasoning fusion.
TCM-adapted AI-HEALS (51)	Implemented disease-specific digital intervention/study	Personalized management of chronic disease multimorbidity	TCM body-constitution assessment and constitution-tailored health-management content	WeChat Mini Program, curated knowledge base, intelligent Q&A, rule-driven targeted content delivery	Three-arm randomized controlled trial protocol; effectiveness results not yet available (51)	Constitution-tailored digital support; effectiveness remains unestablished, and explicit dual-engine fusion is not specified.
SMART Diabetes (9, 52)	Implemented disease-specific digital intervention/study	Primary-care management of type 2 diabetes	No TCM-specific component	Patient/family health promoter mobile tools plus clinical decision support for primary-care clinicians	Cluster-randomized trial protocol (52) followed by a 24-month cluster randomized controlled trial (9)	Clinical trial evidence for digital diabetes management without TCM constitution–syndrome reasoning.
TCM-based mHealth app for prediabetes (53)	Implemented disease-specific digital intervention/study	Prediabetes health management	TCM body constitution and meridian-energy assessment with constitution-based physical-activity and diet advice	Mobile app integrating conventional self-management functions with TCM-specific personalized content	Three-arm randomized controlled trial (*n* = 121) (53)	Trial evidence for TCM-informed prediabetes management; narrower, disease-specific scope.
Current framework	Proposed conceptual architecture (unvalidated)	Longitudinal preventive management across pre-disease and disease-progression-risk stages	Longitudinal constitution-syndrome reasoning under preventive-treatment logic	Digital phenotyping, biomedical risk estimation, fusion, and governed feedback	Conceptual Perspective; integrated system not prospectively validated	Proposed, unvalidated architecture integrating longitudinal dual-engine inference, intervention evidence, clinician oversight, and feedback.

The entries in this table are intentionally heterogeneous in conceptual level and evidence maturity. Conceptual/theoretical frameworks are included as intellectual antecedents, whereas implemented digital interventions are included as empirical comparators. Their inclusion does not imply equivalent evidence status or a head-to-head comparison.

However, the integration process still faces substantial challenges. Future efforts should address interoperable regional data platforms, privacy-preserving data governance, lightweight intelligent equipment, standardized TCM perceptual data, algorithmic bias, clinical safety, and implementation feasibility.

In resource-constrained settings, digitally supported TCM interventions may extend access to structured decision support only if usability, clinical effectiveness, professional workload, and implementation costs are favorable. Affordability, cost-effectiveness, and equity impacts require formal prospective evaluation and should not be inferred from technical scalability alone. The Virtual General Practitioner remains a clinician-supervised decision-support concept, not a substitute for primary healthcare professionals.

This Perspective has several limitations. It is not a systematic review, and the complete architecture has not undergone prospective validation. The supporting evidence is heterogeneous across technologies, diseases, populations, and regions, and several TCM-AI studies report single-center or task-level performance rather than patient outcomes. Clinical safety, algorithmic fairness, implementation burden, and health-economic effects remain incompletely evaluated. In addition, comparator frameworks are evolving rapidly, so the distinctions summarized here will require periodic updating.

A further methodological boundary is that prognostic models and treatment-effect models are not interchangeable. Future validation should assess these components separately, including calibration and external validity for prognostic models and causal validity, treatment-effect heterogeneity, and decision utility for any model used to support intervention selection.

## 9 Conclusion

With further technical maturation, governance, and prospective validation, digitalized preventive treatment may contribute to more continuous and proactive chronic-disease management. The framework proposed here should be viewed as a research agenda rather than an established clinical solution. Rigorous evaluation of feasibility, algorithmic validity, clinical effectiveness, patient-reported outcomes, implementation outcomes, safety, equity, and cost-effectiveness is required before large-scale deployment.

## Data Availability

The original contributions presented in the study are included in the article/Supplementary material, further inquiries can be directed to the corresponding authors.

## References

[1] World Health Organization. Invisible numbers: the true extent of noncommunicable diseases and what to do about them. Geneva: WHO (2022). Available online at: https://www.who.int/publications/i/item/9789240057661 (Accessed October 16, 2026).

[2] Bennett JE Stevens GA Mathers CD Bonita R Rehm J Kruk ME. NCD Countdown 2030 collaborators. NCD countdown 2030: worldwide trends in non-communicable disease mortality and progress towards sustainable development goal target 3.4. Lancet. (2018) 392(10152): 1072–88. 10.1016/S0140-6736(18)31992-5 30264707

[3] World Health Organization. Noncommunicable Diseases Country Profiles 2018. Geneva: World Health Organization, 2018:223. Available online at: https://www.who.int/publications/i/item/9789241514620 (Accessed October 16, 2026).

[4] World Health Organization. World Health Statistics 2023. Geneva: WHO (2023).

[5] Wang Q. Creating an upgraded version of the TCM health engineering for preventing a disease before it arises with nine-constitution identification as the core technology. Tianjin J Tradit Chinese Med. (2019) 36(6):521–7. 10.11656/j.issn.1672-1519.2019.06.01

[6] People's Medical Publishing House (editor). Huangdi Neijing Suwen (Basic Questions of the Yellow Emperor's Internal Classic). Beijing: People's Medical Publishing House, 2012.

[7] Wang Q. TCM Constitutionology. Beijing: People's Medical Publishing House (2021).

[8] Shang HC Zhang XW. Digital-intelligent integration promotes the inheritance and innovative development of traditional Chinese medicine. Beijing J Tradit Chinese Med. (2023) 42(5):464–6. 10.16025/j.1674-1307.2023.05.001

[9] Zhang P Tao X Ma Y Zhang Y Ma X Song H. Improving the management of type 2 diabetes in China using a multifaceted digital health intervention in primary health care: the SMART diabetes cluster randomised controlled trial. Lancet Regional Health Western Pacific. (2024) 49:101130. 10.1016/j.lanwpc.2024.101130 39056088 PMC11269311

[10] Gu TY Yan ZZ Jiang JH. Classifying Chinese medicine constitution using multimodal deep-learning model. Chin J Integr Med. (2024) 30(2):163–70. 10.1007/s11655-022-3541-8 36374441

[11] Zhong R Jiang Y Ni D Yang YL Shi YR Nie SY. Research progress on the analysis process of digital-intelligent TCM (smell) olfaction. J Mathematical Med. (2024) 37(8):561–74. 10.12173/j.issn.1004-4337.202405022

[12] Yip HF Li Z Zhang L Lyu A. Large language models in integrative medicine: progress, challenges, and opportunities. J Evid Based Med. (2025) 18(2):e70031. 10.1111/jebm.70031 40384541 PMC12086751

[13] Tan CYH Koh JYJ Ang WW Tan XM Koh SWC Lin W. State-of-the-art digital phenotyping methods for cardiometabolic risk prevention and management: a scoping review. Int J Med Inform. (2026) 206:106133. 10.1016/j.ijmedinf.2025.106133 41086640

[14] Shang HC. Introduction to Intelligent Traditional Chinese Medicine. Beijing: Science Press (2021).

[15] National Administration of Traditional Chinese Medicine. Several Opinions on Promoting the Development of Digital Traditional Chinese Medicine. Beijing: National Administration of Traditional Chinese Medicine and the National Data Administration of China (2024).

[16] Wang J. Discussion on full-lifecycle health management based on three key issues of TCM constitutionology. J Beijing Univ Tradi Chinese Med (2023) 46(3):377–82. 10.3969/j.issn.1006-2157.2023.03.014

[17] China Association of Chinese Medicine. Guidelines for Intervention on Prevention of Diseases (20 Items). T/CACM 1459-2023 to T/CACM 1477-2023, and T/CACM 1112-2023. Beijing: China Association of Chinese Medicine (2023).

[18] China Association of Chinese Medicine. Guidelines for Intervention of TCM Constitution for the Prevention of Diseases in the Aged People. T/CACM 1459-2023. Beijing: China Association of Chinese Medicine (2023).

[19] Yu Q Wang YH Li ZY Zhang ZL Tian Y Li F. Construction and service of an intelligent TCM health management cloud platform. J Med Inform. (2023) 44(1):54–58, 69. 10.3969/j.issn.1673-6036.2023.01.010

[20] Zhu JJ Zhang Y Gong XH Zheng WY Mao LJ Song HL. Prediction model for elderly diabetic patients complicated with cardiovascular disease based on AI algorithms and TCM constitution types. Shanghai J TraditChinese Med. (2025) 59(3):1–6. 10.16305/j.1007-1334.2025.z20240801001

[21] Duan Y Zhou Q Li Y Qin C Wang Z Kan H. Research on a traditional Chinese medicine case-based question-answering system integrating large language models and knowledge graphs. Front Med (Lausanne). (2025) 11:1512329. 10.3389/fmed.2024.1512329 39839612 PMC11747316

[22] Zhuang Y Yu L Jiang N Ge Y. TCM-KLLaMA: intelligent generation model for traditional Chinese medicine prescriptions based on knowledge graph and large language model. Comput Biol Med. (2025) 189:109887. 10.1016/j.compbiomed.2025.109887 40056842

[23] Beijing University of Chinese Medicine. Wang Qi's team receives the National Science and Technology Progress Award for the establishment and application of the TCM constitution identification system. (2024). Available online at: https://kjc.bucm.edu.cn/xwtp/d66e83ec4b14498495f666079c3bf3ec.htm (Accessed October 16, 2026).

[24] Zhao C Zhang AM Ye ZH Lou J Shi Q Yu Y. A qualitative study on digital-intelligent equipment empowering the “generalized” development of TCM inspection. J Beijing Univ Tradit Chinese Med. (2025) 48(8):1052–61. 10.3969/j.issn.1006-2157.2025.08.003

[25] Zhang M Zhong R Jiang Y Wu ZY Zhang XY Zhao C. Construction and reflections on the digital-intelligent TCM generalized inspection system. J Beijing Univ Tradit Chinese Med. (2025) 48(8):1037–43. 10.3969/j.issn.1006-2157.2025.08.001

[26] Zhou Z Jin D He J Zhou S Wu J Wang S. Digital health platform for improving the effect of the active health management of chronic diseases in the community: mixed methods exploratory study. J Med Internet Res. 2024 26: e50959. 10.2196/50959 39556830 PMC11612601

[27] Qin ZW Yang Y Cen K. Effectiveness evaluation of AI technology applied in TCM health management for patients with type 2 diabetes. Health Res. (2024) 44(6):611–614 + 626. 10.19890/j.cnki.issn1674-6449.2024.06.003

[28] Boima V Doku A Agyekum F Tuglo LS Agyemang C. Effectiveness of digital health interventions on blood pressure control, lifestyle behaviours and adherence to medication in patients with hypertension in low-income and middle-income countries: a systematic review and meta-analysis of randomised controlled trials. eClinicalMed. 2024 69:102432. 10.1016/j.eclinm.2024.102432 PMC10850120 38333367

[29] Wang YZ Li XG Shen QH Wu JL Mo CJ Peng M. Construction and application evaluation of the 5G + AI “three earlies” proactive health management model for chronic diseases. Health Res. 2024 44(6): 606–10. 10.19890/j.cnki.issn1674-6449.2024.06.002

[30] Wang Q. From digits to digits: the past, present and future of TCM digitalization. Digital Chinese Med. 2025 8(01):4– 19. 10.1016/j.dcmed.2025.03.002

[31] Wang X Zhang J Huang Z Wang Y Zhang Y Zhou PP. Construction and application of a “four-in-one” TCM chronic disease management model. Chinese Nur Manag. (2024) 24(11):1608–12. 10.3969/j.issn.1672-1756.2024.11.002

[32] Reicher L Shilo S Godneva A Lutsker G Zahavi L Shoer S. Deep phenotyping of health-disease continuum in the human phenotype project. Nat Med. (2025) 31(9):3191–203. 10.1038/s41591-025-03790-9 40665053

[33] Li X He H Lu G Yue P Chen J Yang Z. TCM-DS: a large language model for intelligent traditional Chinese medicine edible herbal formulas recommendations. Chin Med. (2025) 20(1):191. 10.1186/s13020-025-01249-0 41243110 PMC12621383

[34] Zhang H. Practical dilemmas and compliance responses in healthcare data sharing. Med Philos. (2024) 45(17):52–57. 10.12014/j.issn.1002-0772.2024.17.11

[35] Wen YL Chen MJ. Medical data sharing scheme merging federated learning and blockchain. Comput Eng. (2022) 48(5):145–153, 161. 10.19678/j.issn.1000-3428.0061284

[36] OECD. Health Data Governance for the Digital Age: Implementing the OECD Recommendation on Health Data Governance. Paris, France: OECD Publishing (2022). 10.1787/68b60796-en

[37] Yan WX Hu J Zeng HT Liu M Liang W. Applications and challenges of AI large language models in primary healthcare services. Chinese General Pract. (2025) 28(1):1–6. 10.12114/j.issn.1007-9572.2024.0277

[38] Martínez-Martínez H Martínez-Alfonso J Sánchez-Rojo-Huertas B Reynolds-Cortez V Turégano-Chumillas A Meseguer-Ruiz VA. Perceptions of, barriers to, and facilitators of the use of AI in primary care: systematic review of qualitative studies. J Med Internet Res. (2025) 27:e71186. 10.2196/71186 PMC12242059 40560641

[39] Wang Z Lu J Zhou Z Liu G Zhai X Cao D. Digital health technology adoption among Chinese physicians: latent profile analysis and cross-sectional study. J Med Internet Res. (2025) 27: e77840. 10.2196/77840 41313170 PMC12661596

[40] Hepburn J Williams L McCann L. Barriers to and facilitators of digital health technology adoption among older adults with chronic diseases: updated systematic review. JMIR Aging. (2025) 8:e80000. 10.2196/80000 40934502 PMC12464506

[41] Yang T Wang D Feng MH Huang ZH Duan RX Pu PZ. Research on the Hierarchical Normalization Processing Architecture for Multi-type sensor Data Based on TCM Four Examinations, Western Medicine Tests, and Bioinformatics. World Sci Technol Mod Tradit Chinese Med. 2023 25(2): 798–805.

[42] Luo J Wang XY Zhang JN Li T Bu PZ Yang QC. Discussion on the research status and development trends of intelligent TCM diagnostic equipment. China J Tradit Chinese Med Phar. (2024) 39(10):5386–9.

[43] Li FF Di D Wang YQ Qian P Zheng XY Liu GP. Research on the Information Acquisition and Identification of TCM Facial Inspection Based on Computer Technology. World Sci Technol Mod Tradit Chinese Med. (2008) 10(6): 71–6.

[44] World Federation of Chinese Medicine Societies (WFCMS). Notice of project initiation for the international standard of TCM tongue image data exchange format. SCM NP 2022-0151. (2022).

[45] Watkins DA Msemburi WT Pickersgill SJ Kawakatsu Y Gheorghe A Dain K. NCD Countdown 2030 collaborators. NCD countdown 2030: efficient pathways and strategic investments to accelerate progress towards the sustainable development goal target 3.4 in low-income and middle-income countries. Lancet. (2022) 399(10331):1266– 78. 10.1016/S0140-6736(21)02347-3 35339227 PMC8947779

[46] Zhang X Wei J Liang Y You L Zhang M Shang H. Smart traditional Chinese medicine empowers the whole chain of prevention-screening-diagnosis-treatment-management for major chronic diseases in primary healthcare: research on cardiovascular-cerebrovascular diseases and tumors. Sci Tradit Chinese Med. (2026) 4(2):132–9. 10.1097/st9.0000000000000095

[47] Wu Y Min H Li M Shi Y Ma A Han Y. Effect of artificial intelligence-based health education accurately linking system (AI-HEALS) for type 2 diabetes self-management: protocol for a mixed-methods study. BMC Public Health. (2023) 23:1325. 10.1186/s12889-023-16066-z 37434126 PMC10334542

[48] Wei X Yang X Zhang X Zhao C Jin Y Shang H. Toward complex-systems evidence for Chinese medicine: a four-phase evidence framework. J Evid Based Med. (2026) 19(1):e70111. 10.1111/jebm.70111 41808319

[49] Gou X Gao Z Yang Y Li Q Chen K Lei Y. State-target strategy: a bridge for the integration of Chinese and western medicine. J Tradit Chin Med. (2021) 41(1):1–5. 10.19852/j.cnki.jtcm.2021.01.001 33522191

[50] Wang J Wu Y Jiang Y Bao R Gu X Kong B. Experiences and perceived influence of the artificial intelligence–based health education accurately linking system (AI-HEALS) on health behaviors among patients with type 2 diabetes: qualitative study. J Med Internet Res. (2026) 28:e84605. 10.2196/84605 42118150 PMC13163637

[51] Liu Q Yang Y Hu Y Yang X Liu S Bao R. Study protocol for a randomized controlled trial to evaluate the effectiveness of an artificial intelligence-based health education accurately linking system based on traditional Chinese medicine body constitution in patients with chronic disease multimorbidity. Front Public Health. (2026) 14:1773974. 10.3389/fpubh.2026.1773974 41835430 PMC12982435

[52] Peiris D Sun L Patel A Tian M Essue B Jan S. Systematic medical assessment, referral and treatment for diabetes care in China using lay family health promoters: protocol for the SMARTDiabetes cluster randomised controlled trial. Implement Sci. (2016) 11:116. 10.1186/s13012-016-0481-8 27535128 PMC4989287

[53] Chung HW Tai CJ Chang P Su WL Chien LY. The effectiveness of a traditional Chinese medicine–based mobile health app for individuals with prediabetes: randomized controlled trial. JMIR Mhealth Uhealth. (2023) 11:e41099. 10.2196/41099 37338977 PMC10337399

